# Landscape of *NRXN1* Gene Variants in Phenotypic Manifestations of Autism Spectrum Disorder: A Systematic Review

**DOI:** 10.3390/jcm13072067

**Published:** 2024-04-02

**Authors:** Jaimee N. Cooper, Jeenu Mittal, Akhila Sangadi, Delany L. Klassen, Ava M. King, Max Zalta, Rahul Mittal, Adrien A. Eshraghi

**Affiliations:** 1 Department of Otolaryngology, Hearing Research and Communication Disorders Laboratory, University of Miami Miller School of Medicine, Miami, FL 33136, USA; jcooper12@student.touro.edu (J.N.C.); j.mittal@med.miami.edu (J.M.); asangadi@med.miami.edu (A.S.); dlk123@med.miami.edu (D.L.K.); amk265@med.miami.edu (A.M.K.); 77mrzalta@gmail.com (M.Z.); r.mittal11@med.miami.edu (R.M.); 2 School of Medicine, New York Medical College, Valhalla, NY 10595, USA; 3 Department of Neurological Surgery, University of Miami Miller School of Medicine, Miami, FL 33136, USA; 4 Department of Biomedical Engineering, University of Miami, Coral Gables, FL 33146, USA; 5 Department of Pediatrics, University of Miami Miller School of Medicine, Miami, FL 33136, USA

**Keywords:** *NRXN1*, autism spectrum disorder, gene variants, synaptic function, genotype–phenotype correlation, genetic predisposition

## Abstract

**Background**: Autism spectrum disorder (ASD) is a complex neurodevelopmental condition characterized by social communication challenges and repetitive behaviors. Recent research has increasingly focused on the genetic underpinnings of ASD, with the Neurexin 1 (*NRXN1*) gene emerging as a key player. This comprehensive systematic review elucidates the contribution of *NRXN1* gene variants in the pathophysiology of ASD. **Methods**: The protocol for this systematic review was designed a priori and was registered in the PROSPERO database (CRD42023450418). A risk of bias analysis was conducted using the Joanna Briggs Institute (JBI) critical appraisal tool. We examined various studies that link *NRXN1* gene disruptions with ASD, discussing both the genotypic variability and the resulting phenotypic expressions. **Results**: Within this review, there was marked heterogeneity observed in ASD genotypic and phenotypic manifestations among individuals with *NRXN1* mutations. The presence of *NRXN1* mutations in this population emphasizes the gene’s role in synaptic function and neural connectivity. **Conclusion**: This review not only highlights the role of *NRXN1* in the pathophysiology of ASD but also highlights the need for further research to unravel the complex genetic underpinnings of the disorder. A better knowledge about the multifaceted role of *NRXN1* in ASD can provide crucial insights into the neurobiological foundations of autism and pave the way for novel therapeutic strategies.

## 1. Introduction

Autism spectrum disorder (ASD) is a neurodevelopmental disorder characterized by reduced communication, repetitive behaviors, and restricted interests [1,2,3,4]. ASD can vary in severity from mild to severe. It not only imposes social and personal challenges on the affected individuals and their families or caregivers but also leads to significant financial impacts on them and the healthcare system [5]. Individuals diagnosed with ASD often need comprehensive interventions from an early age, such as physical therapy, occupational therapy, behavioral therapy, medical treatments, specialized education, and assistive technology [6,7]. Additionally, the economic implications are further compounded for those with more severe forms of ASD, who may struggle to achieve financial independence in adulthood.

The prevalence of ASD is gradually increasing leading to a growing economic impact. A study assessed past economic impacts to forecast future financial burdens in the United States at the state level by 2029. Their findings suggest that, if ASD prevalence remains consistent with 2019 levels, the economic impact would reach USD 11.5 trillion by 2029. However, if the prevalence continues to increase as it has in previous decades, the cost could soar to USD 15 trillion [7]. The financial implications of ASD are comparable to those of diabetes and exceed those of stroke and hypertension [6]. The escalating prevalence and substantial economic strain underscore the importance of understanding ASD’s etiology and identifying potential therapeutic targets. While the causes of ASD are multifaceted, genetic factors, combined with possible environmental factors, have been significantly linked to its development [8], with numerous genes potentially associated with it.

The Simons Foundation Autism Research Initiative (SFARI) (https://www.sfari.org/resource/sfari-gene/; Accessed on 15 October 2023) has assembled an open-access evolving database to comprehensively evaluate the role of individual genes in ASD, SFARI Gene. Using stringent and rigorous criteria, SFARI Gene is one of the most reliable databases for the autism research community to delve into the genetic underpinnings of ASD. A key feature of this database is the gene scoring system, where each gene is assigned a rating from 1 to 3, reflecting the robustness of evidence supporting its association with ASD. A designation of 1 indicates genes that have been clearly implicated in ASD with at least three de novo likely gene-disrupting mutations reported, each of which meets a threshold false discovery rate of <0.1. Designations 2 and 3 are genes with two or one reported de novo gene-disrupting mutations, respectively. Genes with the designation 1 are most likely to be truly implicated, while those designated 2 and 3 have an increasing likelihood of being a false positive. In addition, genes can have the designation of S, which means syndromic, indicating these genes carry a substantially increased risk with repeated evidence of their role in ASD. While numerous genes have been identified as having potential links to ASD, establishing a definitive causal relationship presents a greater challenge. SFARI uses an Evaluation of Autism Gene Link Evidence (EAGLE) score designation to help understand the causative relationship of a particular gene with ASD. The higher the EAGLE score for a gene, the higher the probability of it being causative in ASD. For genes with an EAGLE score of 12 or higher, their causative role in ASD is supported by consistent and repeated evidence from both research and clinical settings, and this relationship has been demonstrated over time. A designation of 12 and over also means there are no published papers that contradict its role in ASD. In this review, we have focused on *NRXN1*, which is a category 1 gene with the highest EAGLE score of 146. *NRXN1* has been repeatedly and consistently shown to be strongly associated with ASD [9,10,11,12,13,14,15,16,17].

**Figure 1:**
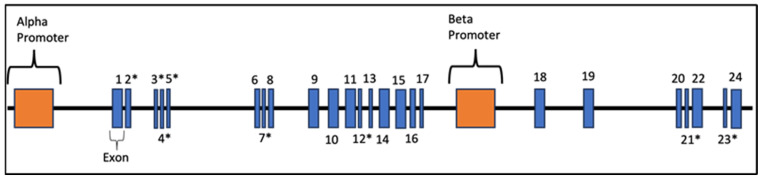
*NRXN1* gene structure. A schematic illustrating the organization of *NRXN1* gene. Exons are depicted as blue boxes and numbered. Asterisks indicate exons where alternative splicing occurs. This image indicates the relative size and location of its exons and introns, as well as promoters. * indicates alternative splice site.

**Figure 2:**
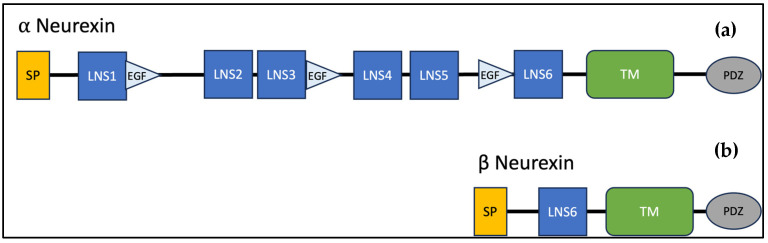
Structural domain organization of both alpha and beta isoforms of neurexin. (**a**). Alpha neurexin. (**b**). Beta neurexin. SP, signal peptide; LNS, laminin/neurexin/sex hormone binding domain; EGF, epidermal factor-like region; TM, transmembrane domain; PDZ, PSD-95, DLG1, ZO-1 domains.

Increasingly, there is evidence that dysfunction at the level of the synapse is central to the pathophysiology of ASD [18,19,20,21,22,23,24,25]. *NRXN1*, located at 2p16.3, encodes neurexins, which are a family of presynaptic cell adhesion molecules that are central to creating and modifying synaptic connections and have been implicated in the pathophysiology of ASD and other neuropsychiatric disorders (Supplemental Figure S1) [8,11,13,25,26,27,28,29,30,31,32,33,34,35,36,37,38]. Neurexins are encoded by three genes (*NRXN1, NRXN2, NRXN3*), each of which contain two promoters, one for a longer alpha isoform and the other for a shorter beta isoform (Figure 1). The first promoter is located upstream of exon 1 and allows for transcription of alpha isoforms and the second promoter is located medially, between exon 17 and 18, to code the beta isoforms. These isoforms undergo extensive alternative splicing yielding over 1000 neurexin mRNAs. *NRXN1* and *NRXN3* are two of the largest mammalian genes. *NRXN1* is 1.12 Mb long containing 24 exons, and multiple splice sites [8,39,40,41,42]. The translated protein is a transmembrane protein, which contains an extracellular domain, a transmembrane region, and an intracellular motif that controls downstream events (Figure 2). Alpha neurexin is comprised of six extracellular domains with three epidermal growth factor (EGF)-like regions [13,43,44]. Beta neurexins are smaller and contain only one extracellular domain. Both alpha and beta neurexins have been implicated in the pathophysiology of ASD [12,45,46,47]. The precise structure of neurexins has yet to be fully elucidated in humans but has been modeled from other mammals such as mice and rats.

*NRXN1* is essential for synaptic health in central nervous system (CNS) and peripheral nervous system (PNS) activity. It has been implicated in various neuropsychiatric conditions including ASD, attention deficit hyperactivity disorder (ADHD), seizures, schizophrenia, psychosis, and bipolar disorder [9,48,49,50,51,52,53]. *NRXN1* plays a major role in the differentiation of synapses and is found in high concentrations in the supporting cells of the brain [54,55,56,57,58,59]. Deficits in *NRXN1* have been associated with intellectual/learning disability and speech/language/global development delay, which are some of the hallmarks of ASD [12,29,45,60,61,62,63,64,65]. Moreover, research shows that children who harbor *NRXN1* gene variants have behavioral problems such as aggression and tantrums, as well as balance and motor skill deficits [66]. These phenotypic expressions are consistent with those commonly observed in some individuals with ASD, providing important insights that could enhance our understanding of the relationship between ASD and *NRXN1* mutations.

NRXN1 interacts with protein structures called Neuroligins. Neuroligins are postsynaptic molecules that bind with NRXNs to create a calcium-dependent complex [67,68]. This complex is crucial for neurotransmission and allows for the recruitment of neurotransmitter receptors as well as other structural proteins required for effective neurotransmission. Precise control over excitation and inhibition at the level of the synapse is vital for the appropriate function of the nervous system, with alterations having been linked to ASD pathology. Mutations in *NRXN1* and other associated gene regions have been linked to both reduced and increased excitatory synaptic activity as well as decreased neurotransmitter release [69]. Notably, ASD is related to an imbalance of excitation and inhibition at the level of the synapse; however, the exact mechanism and direction of the imbalance is still under investigation [70].

The objective of this systematic review article is to comprehensively review the role of *NRXN1* gene variants in the manifestations of ASD as well as to analyze the phenotypic outcomes. Enhancing our knowledge of the involvement of *NRXN1* gene variants in the pathophysiology of ASD holds important clinical implications. This knowledge would facilitate the creation of tailored genetic counseling for affected individuals, informing patients as well as families and caregivers of more individualized disease outcomes and expectations. Additionally, this understanding could enable the development of effective treatment approaches, leveraging advanced genomic editing technologies such as CRISPR/Cas9 as well as more traditional drug regiments [71,72,73,74,75].

## 2. Materials and Methods

### 2.1. Search Strategy

This systematic review was conducted in accordance with the Preferred Reporting Items for Systematic Reviews and Meta-Analyses (PRISMA). The protocol for this systematic review was designed a priori and was registered in the PROSPERO database (registration number: CRD42023450418). A literature search was performed in PubMed, Embase, Web of Science, and SCOPUS databases using the MeSH terms; “*NRXN1*” AND “autism”; “Genetic impact of *NRXN1*” AND “autism”; “*NRXN1* in ASD”; “*NRXN1*-associated autism”; “Neurexin 1 variants” AND “ASD clinical outcomes”; “Functional implications of *NRXN1*” IN “autism spectrum disorders”; and “*NRXN1* and ASD/autism phenotypic manifestations”.

### 2.2. Study Selection

At least three reviewers (A.K., D.K., and J.C.) independently reviewed all searched articles, abstracts, and full-text publications. Any disagreements on the exclusion or inclusion of results were resolved by a fourth reviewer or senior author. All included studies required a diagnosis of ASD by gold-standard diagnostic criteria such as DSM 5, ADOS-2, and ADI. Study inclusion criteria included participant diagnosis of ASD at any age and publication between November 2018 and September 2023, as there were previous reviews available that updated the literature until November 2018. Study exclusion criteria included no formal ASD diagnosis, publication not in a peer-reviewed article, reviews, commentaries, conference proceedings, case reports/studies, non-human studies, ex-vivo or in-vitro studies, and studies not originally published in English.

### 2.3. Data Extraction

Three investigators (A.S., A.K., D.K., and J.C.) independently reviewed included articles. The information gathered includes: study type, population, comparison/study, ASD diagnosis criteria, outcomes/conclusions. After initial data extraction, each investigator gathered data on the zygosity, specific mutation, inheritance, intellectual abilities, speech abilities, physical characteristics, behavioral diagnoses, consanguinity, and gender.

### 2.4. Quality Assessment

A Risk of Bias (RoB) analysis was conducted using the Joanna Briggs Institute (JBI) Critical Appraisal Tool. The appropriate checklist was utilized based on the type of study. This assessment was completed by four reviewers (A.K., D.K., A.S., and J.C.) independently, with discrepancies resolved by discussion and consensus or discussion with the senior author.

## 3. Results and Discussion

**Figure 3:**
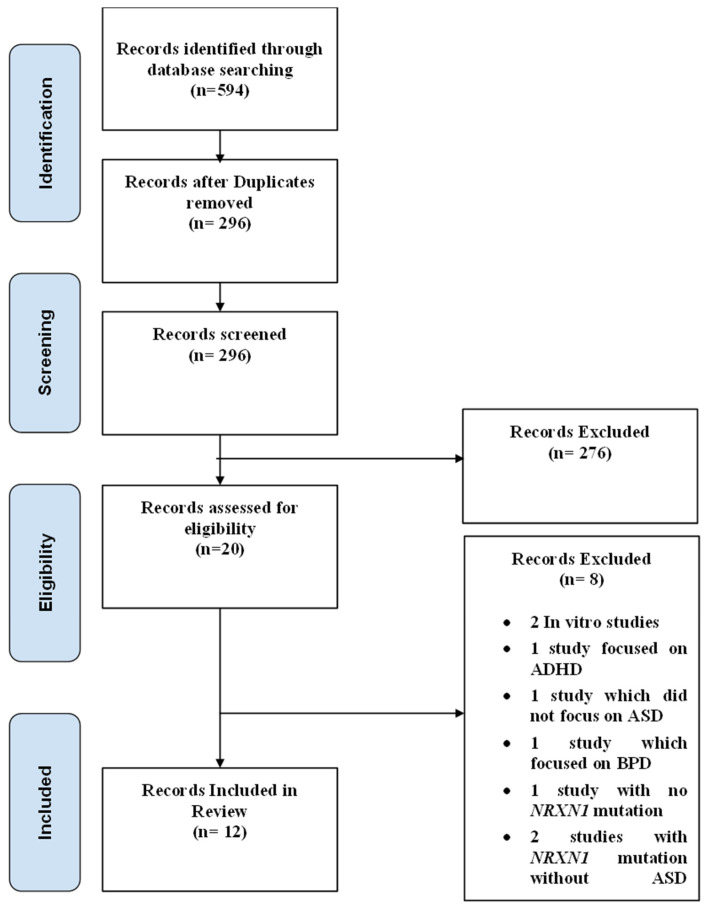
PRISMA flow diagram for study selection: This figure represents a PRISMA (Preferred Reporting Items for Systematic Reviews and Meta-Analyses) flow diagram, showing the detailed process of study selection for this systematic review.

**Figure 4:**
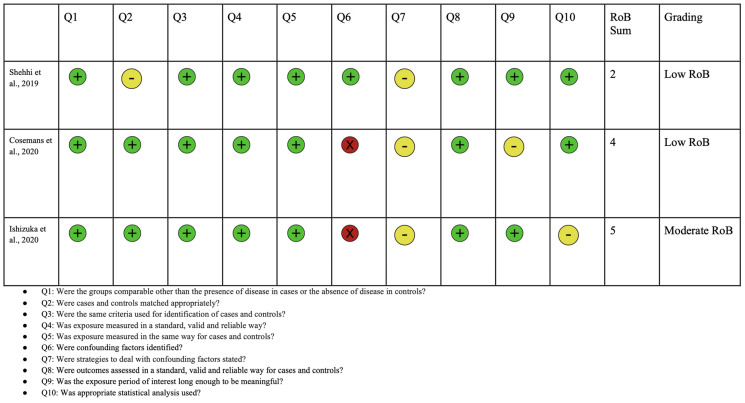
Risk of bias analysis using Joanna Briggs Institute for case control studies. Green circle represents low bias, yellow circle represents unclear, and red indicates high bias [60,76,77].

**Figure 5:**
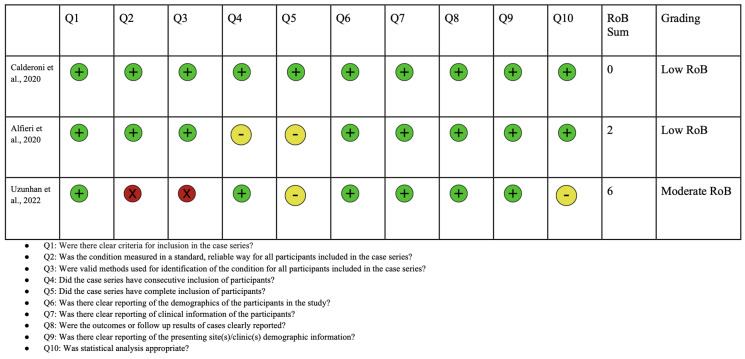
Risk of bias analysis using Joanna Briggs Institute for case series studies. Green circle represents low bias, yellow circle represents unclear, and red indicates high bias [78,79,80].

**Figure 6:**
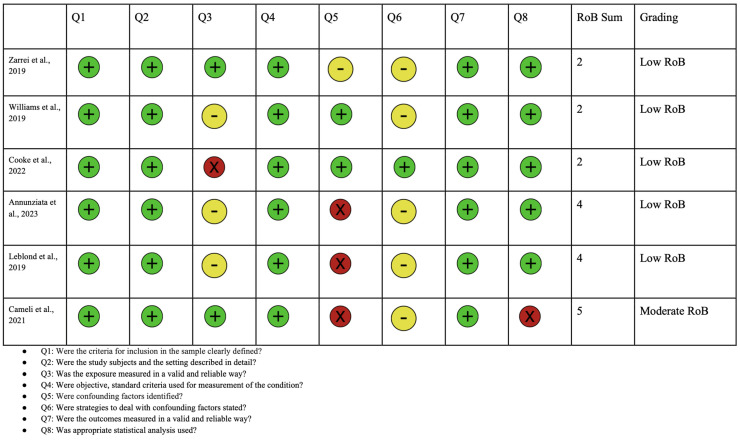
Risk of bias analysis using Joanna Briggs Institute for cross sectional studies. Green circle represents low bias, yellow circle represents unclear, and red indicates high bias [27,36,81,82,83,84].

A PRISMA diagram showing the criteria for included studies in this systematic review article is shown in Figure 3. Following the initial search, a total of 594 studies were identified. Through screening, 296 records were selected excluding duplicates and irrelevant articles. After conducting a full-text review, 12 articles were chosen for inclusion in this study. This selection was made after excluding studies for reasons detailed in the PRISMA diagram, including irrelevance of outcomes to this study or to ASD. Each of the included articles provided data on mutations in the *NRXN1* gene and their association with ASD. A risk of bias analysis was performed using the JBI critical appraisal checklist. Risk of bias analyses for case control, case series, and cross-sectional studies are shown in Figure 4, Figure 5, and Figure 6, respectively. Overall, the studies included in this systematic review were found to have a low risk of bias and were deemed to be of suitable quality for inclusion.

### 3.1. Patient Population and Diagnosis

The total number of individuals with ASD included in this study is 2247, with a subset of 71 participants found to also carry a mutation in NRXN1. Of those with both ASD and an NRXN1 mutation 57.8% were male and 21% were female (21% of the cohort had no gender specified in the study). The diagnostic methods used for ASD in each study has been summarized in Table 1.

### 3.2. Genotypic Variants of NRXN1 in Individuals with ASD

As genetic sequencing becomes more affordable and accessible, delving into the genetic underpinnings of variable clinical presentations has been at the forefront of research. In line with this, each included study in our review conducted genetic testing on groups of individuals with ASD to explore genetic factors. The estimated rate of *NRXN1* variants in the general population is thought to be around 0.21% [85]. The prevalence of NRXN1 mutation in this population is 3.1% of all patients included who had a diagnosis of ASD.

In all studies, the parents of affected children who also harbored *NRXN1* mutations were found to be undiagnosed with ASD, thereby serving as carriers of the genetic variations. This finding highlights the multifaceted nature of ASD and the idea that multiple factors occurring together may be needed for the phenotypic clinical expression. The predominant trend observed was exonic deletion mutations [49,63,71,72,73,74,75,85]. An overview of genotypic information is shown in Table 2. Exons are segments of the gene that are translated into proteins, thereby directly affecting synaptic health and function. This finding is particularly significant given that *NRXN1* expression is predominantly found in the CNS and reaches its highest expression levels during periods of critical neurodevelopment [8,86]. As previously stated, the two main isoforms for *NRXN1* are a longer alpha isoform and a shorter beta isoform. Six of the included studies observed that the alpha isoforms were affected, comprising 20 individuals, and two studies found the beta isoform to be affected across three individuals [78,80,83]. There is a low penetrance of *NRXN1* mutations, as evidenced by the lack of ASD manifestation in all parental or sibling carriers involved in these studies as well as data from previous studies [87]. However, it appears that these mutations still confer a risk for ASD development when combined with other genetic factors or exposure to environmental factors such as environmental toxins, drugs, pollution, or gestational/perinatal events. In the studies reviewed, three reported participants with heterozygous mutations, one observed a homozygous mutation, and six did not specify the mutation status of the patients. Previously, heterozygous *NRXN1* mutations have been associated with ASD among other reported neuropsychiatric disorders [46]. Overall, deletions comprised a majority of the mutations described in these studies. Given the observed association between *NRXN1* mutations and ASD, these genetic modifications are considered crucial in understanding not only ASD but also other neuropsychiatric and behavioral disorders. Therefore, a deeper examination of the connection between the *NRXN1* gene and ASD is vital to comprehend how this gene influences the ASD phenotype and its prognosis. Table 3 has an overview of the mutations found in each patient from the included studies.

This review focuses on the mutations found within *NRXN1* and its subsequent phenotypic and genotypic profiles. When examining the included articles an unexpected finding was the role of non-coding RNA regions, which affected the expression of genes. These were discussed by Williams et al., Zarrei et al., and Annunziata et al. While not a focal point of this review, these adjacent mutations are important to discuss. Williams et al. found that SNV in miR-873-5p affect the expression of 109 SFARI candidate genes. Zarrei et al. and Annunziata et al. found mutations that disrupted the transcription of AK127244 (LOC730100), which is a non-coding RNA of unknown function adjacent to *NRXN1* [81,84]. Annunziata et al. also found a mutation in a small segment distal to NRXN1 that similarly impacted the functioning of the gene products. This emerging field of study involving the pathological effects of long non-coding RNAs (lncRNAs) is gaining interest, especially since they are more abundant in the human brain when compared to protein-coding RNAs [88]. This abundance hints at their potential role in neuropsychiatric diseases [77]. lncRNAs also play a critical role in the normal functioning of various physiological systems in humans and are associated with various disease states, including cancers, neurological disorders, and cardiovascular diseases [83]. AK127244 is associated with deletions in 2p16.3, which is located within close proximity to *NRXN1* and has been noted to induce similar clinical characteristics as mutations directly affecting *NRXN1* [89,90]. These mutations are not, on their own, causative of ASD. However, in conjunction with another mutation, such as those from the SFARI database, they play a role in expression of the ASD phenotype.

Consanguinity is often investigated in relation to genetic disorders, particularly those that are transmitted recessively across generations, such as *NRXN1*. When two individuals who are closely related have children, it increases the occurrence of recessive genetic disorders when compared with unrelated pairings. Uzunhan et al. studied two specific cases—two siblings with consanguineous parents, each with an *NRXN1* deletion. One sibling had a diagnosis of West syndrome while the other had a diagnosis of ASD. West syndrome is an encephalopathy that presents as infantile spasms and may include neurodevelopmental delays/regression. It should be noted that up to 20% of people with West syndrome have a diagnosis of ASD at some point in their lifetime [80,91,92,93]. CNV analyses of these patients found an *NRXN1* exon 2–5 homozygous deletion that affected the alpha-isoform in both siblings. This mutation was confirmed with gel electrophoresis and compared with four healthy control individuals.

The genotypic findings of these articles are non-uniform and cannot be used to make conclusive relationships between *NRXN1* mutations and ASD. Many included articles had only a handful of participants with both ASD and *NRXN1* mutations and contain inconsistent reporting of genetic information across studies. Further studies having large cohorts are warranted to decipher the precise role of *NRXN1* mutations in the pathophysiology of ASD.

### 3.3. Phenotypic Features of NRXN1 Mutations and ASD

#### 3.3.1. Intellectual Abilities

The phenotype of individuals harboring *NRXN1* variants is vast and heterogenous. ASD is known to affect males at a rate almost four times higher than for females, which is reflected in this cohort [94]. Intellectual abilities were recorded in six studies, 24% of participants with both ASD and a *NRXN1* mutation were observed to have an intellectual disability and 14% were found to have no intellectual disability as measured by a standard questionnaire. The remaining 62% of participants had no recording of intellectual ability or were unable to complete the required tests for a determination of intellectual ability, which is in line with previous findings of diagnosed intellectual disability in those with *NRXN1* mutations [66].

#### 3.3.2. Speech Abilities

One major challenge was the lack of consistent reporting on both genotypic and phenotypic data from all included articles, many primarily focused on the genetic or phenotypic aspects of ASD, with minimal emphasis on the alternative aspect. In the included individuals with ASD and an *NRXN1* mutation, 2.7% were verbal, 5.5% were non-verbal, and 29% were designated as having a speech delay; the remainder had no recorded information on verbal abilities [60,78,79,82,95].

#### 3.3.3. Behavior/Neuropsychiatric Diagnosis

Many of the participants had behavioral problems including aggression and attention deficits. Alfieri et al. found two of their three individuals with ASD to be aggressive and Ishizuka et al. found one patient with a diagnosis of oppositional defiant disorder. Al Shehhi et al. and Ishizuka et al. had participants with diagnosed ADHD. Cameli et al. found their participant with ASD and an *NRXN1* mutation to have hyperactive behaviors, but no confirmed ADHD diagnosis. The remainder of the studies did not collect data on comorbid ADHD, or it was not reported. These behavioral findings coincide with prior studies that found *NRXN1* to be associated with aggression and attention deficits in animal models [96,97].

Cosemans et al. evaluated the association between having a diagnosis of a neurodevelopmental or neuropsychiatric condition and possessing the *NRXN1* deletion [76]. This study used two cohorts of patients: a literature cohort of 670 individuals and a Leuven cohort of 43 individuals. Controls included patients who were screened for intellectual disability, developmental disorder, ASD, or schizophrenia. In this study, they found that individuals in the literature cohort who did not have a confirmed neurodevelopmental and neuropsychiatric condition were less likely to have the *NRXN1* deletion than those who had the phenotype. The authors suggested that this relationship had concerns about reporting bias as the phenotypes associated with intrinsic patients are not commonly reported. Two exon deletions were analyzed: exons 1–5 and 5–24. The deletions for exon 1–5 was seen in 20 controls (12.59 penetrance) and the deletions for exon 6–24 was seen 2 times in 100,000 individuals (32.43 penetrance). In patients who were diagnosed with intellectual or developmental disability, 133/260 had deletions in the *NRXN1* gene. Deletions were seen in 245/458 individuals with a psychiatric diagnosis. Additionally, psychiatric diagnoses were seen the most with deletions in exon 1- 5 and intron 5. Deletions in exons 6–24 had the greatest association with diagnoses of intellectual and developmental disability (43/68). Our analysis only included that of the Leuven cohort as these data are previously unanalyzed. In the Leuven cohort, they found that patients with de novo deletions of exons 1–5 had phenotypes of intellectual disability and autism. Patients with a maternally inherited deletion of exons 1–5 had ASD with some intellectual disability and psychiatric symptoms (delusions and psychotic episodes). Patients with maternally inherited deletions of exons 6–9 had developmental delay, hypertelorism, and inner epicanthal folds. Patients with a de novo exon 19 deletion had ASD, intellectual disability, and anxiogenic and behavioral deficits. These NRXN1 deletions were also seen in 143/258, 73/144, 63/125, and 44/88 individuals with ASD, schizophrenia, congenital abnormalities, and epilepsy, respectively. This study serves as an outstanding model for integrating genotypic as well as phenotypic information and should be a guiding reference for future analyses involving *NRXN1* and ASD.

#### 3.3.4. Physical Characteristics

Physically, there were no recurrent abnormalities observed to be present across the included cohorts. Macrocephaly or relative macrocephaly was noted in two participants and facial dysmorphia in four. There are single incidences of physical abnormalities such as pyloric stenosis, sensorineural hearing loss and pectus excavatum but no incidences of repeated patterns of these disorders. Prior studies have noted the presence of epilepsy in those with ASD and *NRXN1* mutations. Eight participants were noted to have seizures but no documented epilepsy diagnoses. There were no incidences of brain, cardiac, or urogenital abnormalities in the included cohort.

Many of the participants in this review had comorbid motor and behavioral symptoms; however, the precise symptoms varied greatly. A comprehensive overview of the phenotypic manifestations reported in the included studies along with patient population summary and diagnosis has been shown in Table 4. Expanding the scope of research to include larger studies that meticulously document all phenotypic information of the participants could lead to a more comprehensive understanding of phenotypic patterns. These studies, by encompassing a broader range of phenotypic data, can provide deeper insights into variations and commonalities among individuals. This in turn might reveal subtler correlations or trends that smaller studies might miss. By gathering a comprehensive range of phenotypic information, researchers are able to more effectively identify and understand the subtleties within these patterns, potentially leading to more targeted and effective interventions or therapies.

This review provides insights into the genotypic and phenotypic outcomes observed in the included studies. It reveals significant diversity in both the genetic variants and physical characteristics of individuals harboring the *NRXN1* gene variants in line with previous findings [98]. There is speculation that the size and the location of the *NRXN1* mutation may influence the phenotypic presentation of autism [27]. Large studies with a dual focus on genotype and phenotype are essential to understanding the relationship of *NRXN1* mutations with its various clinical presentations.

## 4. Limitations

A significant limitation of our review stems from the limited pool of participants exhibiting both ASD and *NRXN1* genetic variations within the studies we have analyzed for this article. The dearth of individuals with this specific combination of traits may impact the generalizability and reliability of our findings. The small sample size not only hinders statistical power but also raises concerns about the applicability for a broader population. It is essential to acknowledge this limitation as it highlights the need for further research with more extensive and diverse cohorts to establish more robust conclusions regarding the relationship between ASD and *NRXN1* variations. Additionally, there were inconsistencies in the information reported in the included studies. The articles predominantly focused on either the genotype or phenotype, offering limited information on the other aspect. To compensate for this, the reviewers had to rely on Supplemental Information, especially for articles centered on genotypes, to gain a clearer understanding of the phenotypic outcomes. Ultimately, more comprehensive research involving individuals with ASD is necessary to better understand the relationship between *NRXN1* gene variants and phenotypic expression.

## 5. Conclusions and Future Directions

This systematic review article delves deep into the phenotypic manifestations of ASD in individuals carrying *NRXN1* gene variants. The included studies suggest heterogeneity in both the genotype and phenotype of individuals harboring *NRXN1* gene variants. While the genotype and phenotype associated with *NRXN1* is not fully elucidated, this review continues to shed light on the implication of this mutation on neuropsychiatric disorders, including ASD. Our findings were consistent with prior literature, which found exonic mutations to be commonly associated with neuropsychiatric disease and *NRXN1* mutations [99], as well as found a low prevalence of dysmorphic features and a relatively high frequency of seizure disorders.

Although there have been advances in understanding the role of *NRXN1* in ASD, there remain major research gaps. There is a need for more in-depth studies to elucidate the precise mechanisms by which *NRXN1* gene variants contribute to the development and manifestation of ASD. Understanding the biological pathways involved is essential for targeted therapeutic strategies. Research has not fully explored the range of phenotypes associated with different *NRXN1* mutations in ASD. Further studies are needed to clarify the relationship between specific *NRXN1* variants and the spectrum of autism symptoms. In addition, the interaction between *NRXN1* gene variants and environmental factors in the development of ASD is not well understood. Research in this area could provide insights into potential triggers or protective factors. Furthermore, much of the current research has been conducted in limited populations. There is a gap in studies involving diverse ethnic geographical, and gender populations, which is crucial for understanding the global impact of *NRXN1* variants on ASD. Furthermore, there is a lack of long-term, longitudinal studies following individuals with *NRXN1* gene variants from early childhood into adulthood. Such studies could offer valuable insights into how these variants influence the progression and outcomes of autism over time.

Despite these research gaps, *NRXN1* gene variants have been strongly associated with ASD. Thus, focusing on this specific gene could enhance the prognosis of ASD. It is well-known that treating genetic disorders is a complex challenge, typically addressed by a comprehensive, multi-faceted team employing various therapeutic modalities. One of the emerging approaches in treating genetic disorders involves gene editing [100,101,102]. This technique uses a biological system, such as CRISPR/Cas9, to precisely excise a specific gene segment and employ a biological vector to insert a desired sequence [103,104,105,106]. These treatments are ideal, as they are permanent, unlike the multimodal management that is currently being utilized [107]. Identifying likely genetic etiologies of ASD is the first major step in creating gene editing for this disease. In addition to genetic therapies, targeted drug therapies for *NRXN1* related molecules may be useful in treating the associated clinical symptoms [108]. These therapies include RNAi, protein replacement, small molecule therapy, and chaperone therapy [109,110,111,112,113,114].

While ASD is believed to be influenced by multiple factors, *NRXN1* stands out as a well-researched genetic factor in ASD. With an EAGLE score of 143.75, it has consistently been linked to, and potentially implicated in, the development of ASD. This positions it as an ideal candidate for gene-editing technologies. Conducting genetic testing in individuals with ASD, along with in-depth studies on the phenotypic effects of *NRXN1* mutations, can enhance our understanding of how *NRXN1* contributes to ASD. By directly targeting this gene, we could pave the way for developing innovative therapeutic approaches, potentially leading to improved treatment outcomes and prognoses for ASD in the future in pursuit of improving quality of life of affected individuals.

## Figures and Tables

**Table 1:** Diagnostic tests for ASD.

Study	Diagnostic Test for ASD
Alfieri et al. [78], 2020	ADOS2
Annunziata et al. [81], 2023	DSM5
Calderoni et al. [79], 2020	DSM 5
Cameli et al. [82], 2021	ADOS
Cooke et al. [27], 2022	DSM 5 or ICD
Cosemans et al. [76], 2020	DSM 5
Ishizuka et al. [77], 2020	DSM 5
Leblond et al. [36], 2019	ICD-10 criteria for childhood autism/autistic disorder Gillberg criteria for Asperger syndrome ICD-10 criteria for atypical autism with the added requirement that a case thus diagnosed could not meet full criteria for childhood autism or Asperger syndrome ICD-10 criteria for disintegrative disorder
Shehhi et. Al. [60], 2019	Gold standard test—unspecified
Uzunhan et al. [80], 2022	Gold standard test—unspecified
Williams et al. [83], 2019	DSM 4
Zarrei et al. [84], 2019	ICD11 or DSMV

**Table 2:** An overview of genotypic information.

Reference	Total ASD Cases	Total ASD and NRXN1 Nutation Cases	Mutation in Alpha Isoform	Mutation in Beta Isoform	Unspecified Mutated Isoform	Number of Exonic Deletion	Number of Intronic Deletions	Frequency of Exonic Deletion	Frequency of Intronic Deletion	Homozygous Mutation	Heterozygous Mutation
Alfieri et al. [78], 2020	5	3	NR	NR	3	0	0	NR	NR	0	3
Annunziata et al. [81], 2023	209	3	NR	NR	3	0	0	NR	NR	NR	NR
Calderoniet al. [79], 2020	93	2	NR	NR	2	0	0	NR	NR	NR	NR
Cameli et al. [82], 2021	104	1	1	1	0	1	0	1	0	NR	NR
Cooke et al. [27], 2022	69	12	NR	NR	12	0	0	NR	NR	NR	NR
Cosemans et al. [76], 2020	43	17	NR	NR	17	0	0	NR	NR	NR	NR
Ishizuka et al. [77], 2020	192	5	3	0	2	3	0	0.6	0	0	5
Leblond et al. [36], 2019	36	1	1	0	0	1	0	1	0	NR	NR
Shehhi et. Al. [60], 2019	20	20	13	2	5	13	7	0.65	0.35	NR	NR
Uzunhan et al. [80], 2022	1	1	1	0	0	1	0	1	0	1	0
Williams et al. [83], 2019	48	1	1	0	0	0	0	NR	NR	0	1
Zarrei et al. [84], 2019	1838	6	NR	NR	6	6	0	1	0	NR	NR

NR: not recorded.

**Table 3:** Mutations in the review population.

Reference	Mutation Location
Alfieri et al. [78], 2020	arr[GRCh37] 2p16.3(50432664_50536137)x1 mat, arr[GRCh37] 2p16.3(51086847_51411126) x1 mat, arr [GRCh37] 2p16.3(51037104_52339655)x1 pat.
Annunziata et al. [81], 2023	arr[GRCh37/hg19] 2p16.3(51066578_51100412)x1, arr[GRCh37/hg19] 2p16.3(51175725_51328842)x1 mat, arr[GRCh37/hg19] 2p16.3(50039172_50735499)x1 mat,
Calderoni et al. [79], 2020	arr[GRCh37/hg19] 2p16.3 (50909765_51083469) 1x pat
Cameli et al. [82], 2021	2p16.3 (NC_000002.11:g.50170766_50982172del)
Cooke et al. [27], 2022	unspecified from SynaG cohort
Cosemans et al. [76], 2020	arr[GRCh37/hg19] 2p16.3 (50453695_50662935), arr[GRCh37/hg19] 2p16.3 (50879191_50953066) mat, arr[GRCh37/hg19] 2p16.3 (50620243_50970739), arr[GRCh37/hg19] 2p16.3 (51017528_51302432) mat, arr[GRCh37/hg19] 2p16.3 (50898653_51104632) pat, arr[GRCh37/hg19] 2p16.3 (50923553_51034676) pat, arr[GRCh37/hg19] 2p16.3 (51033135_51074619), arr[GRCh37/hg19] 2p16.3 (51033989_51062766), arr[GRCh37/hg19] 2p16.3 (50992089_51026709), arr[GRCh37/hg19] 2p16.3 (51027631_51390231), arr[GRCh37/hg19] 2p16.3 (51039779_51297569) mat, arr[GRCh37/hg19] 2p16.3 (50497204_50514746) mat, arr[GRCh37/hg19] 2p16.3 (50497204_50514746) mat, arr[GRCh37/hg19] 2p16.3 (51053925_51319222) mat, arr[GRCh37/hg19] 2p16.3 (50975806_51005275), arr[GRCh37/hg19] 2p16.3 (51063155_51278187), arr[GRCh37/hg19] 2p16.3 (51160878_51356269) pat
Ishizuka et al. [77], 2020	rs201336161, rs201881725, rs1457374261, rs199970666
Leblond et al. [36], 2019	del(2p16:51125625-51255427)
Shehhi et. Al. [60], 2019	Del(2p16.3:50,138,031–50,214,776), Del(2p16.3: Del(2p16.3:50,138,031–50,214,776), Del(2p16.3: Del(2p16.3: 50,483,652–50,495,891), Del(2p16.3: Del(2p16.3: 50,483,652–50,495,891), Del(2p16.3: Del(2p16.3: 50,690,984–50,870,064), Del(2p16.3: Del(2p16.3: 50,881,995–50,947,729), Del(2p16.3: Del(2p16.3: 50,947,670–50,964,907), Del(2p16.3: Del(2p16.3: 50,957,455–51,251,557), Del(2p16.3: Del(2p16.3: 50,964,848–51,251,557), Del(2p16.3: Del(2p16.3: 50,968,453–51,260,612), Del(2p16.3: Del(2p16.3: 50,982,113–51,446,873), Del(2p16.3: Del(2p16.3: 51,057,824–51,142,908), Del(2p16.3: Del(2p16.3: 51,083,410–51,172,182), Del(2p16.3: Del(2p16.3: 51,122,091–51,314,430), Del(2p16.3: Del(2p16.3: 51,122,091–51,382,872), Del(2p16.3: Del(2p16.3: 51,122,091–51,606,257), Del(2p16.3: Del(2p16.3: 51,137,071–51,314,430), Del(2p16.3: Del(2p16.3: 51,148,508–51,251,557), Del(2p16.3: Del(2p16.3: 51,153,052–51,260,612), Del(2p16.3: Del(2p16.3: 51,237,000–51,260,612)
Uzunhan et al. [80], 2022	Del(2p16.3:chr2:51149007–51255411)
Williams et al. [83], 2019	chr2:50847195; rs78540316
Zarrei et al. [84], 2019	Del(2p16.3:50,138,031–50,996,179)pat, Del(2p16.3: 50,986,743–51,644,735), Del(2p16.3: 51,125,058–51,263,149), Del(2p16.3: 51,141,571–51,363,855)pat, Del(2p16.3: 51,163,235–51,285,498)pat, Del(2p16.3: 51,163,990–51,285,498)pat

**Table 4:** A comprehensive summary of included studies in this systematic review.

Reference	NRXN1 Isoform Effected	Other Molecular Findings	Parental Consanguinity	Family History	Developmental Delay	Intellectual Disability	Seizures	EEG	Motor Abnormalities (Movement, Speech)	Sensory abnormalities (Hearing, Vision)	Behavioral Abnormalities	Other
Calderoni et al. [79], 2020. Case 1	NR	NR	NR	NR	NR	Normal IQ (>70)	NR	NR	Verbal/non-verbal	none	none	n/a
Calderoni et al. [79], 2020. Case 2	NR	duplication at Xp22.33	NR	NR	NR	Low IQ (<70)	NR	NR	none	none	none	n/a
Alfieri et al. [78], 2020. Case 1	NR	None	NR	NR	NR	Below average on TDQ (30)	-	-	Hypotonia, no speech	none	Tantrums, aggression, self-injurious behavior	Trichotillomania, teeth grinding
Alfieri et al. [78], 2020. Case 2	NR	None	NR	NR	NR	Below average TDQ (44)	-	-	Chewing difficulties only babbles	none	none	Smoking and medication exposure in utero
Alfieri et al. [78], 2020. Case 3	NR	None	NR	NR	NR	Below average NVIQ (74)	+	-	Motor dysregulation	none	Attention problems	Multiple ear infections
Alfieri et al. [78], 2020. Case 4	NR	None	NR	NR	NR	Below average FSIQ (50)	-	-	none	none	Paranoid ideation, aggressive behavior	allergies, sIgA deficiency, recurrent respiratory infections
Alfieri et al. [78], 2020. Case 5	NR	None	NR	NR	NR	Below average NVIQ (72)	n/a	n/a	none	soliloquy	Shy, withdrawing, avoidant behavior	IUGR, sleep problems
Zarrei et al. [84], 2019	NR	AK12724	NR	NR	NR	NR	NR	NR	NR	NR	NR	NR
Cooke et al. [27], 2022	Alpha, beta, theta	n/a	n/a	ASD, ADHD, anxiety, depression	yes	Below average to average	no	yes	Eye movements/gaze patterns	no	Repetitive and restrictive behaviors	n/a
Cosemans et al. [76], 2020	beta	n/a	n/a	ASD, psychiatric problems, intellectual disability, IQ	yes	yes	no	no	Repetitive movements	no	Anxiety behaviors	n/a
Shehhi et. Al. [60], 2019	Alpha and beta	n/a	n/a	Congenital heart disease, global development delay, epilepsy, intellectual disability, speech delay	yes	yes	Hallucinations, yes in some cases	yes	Gross motor delay	Sensorineural hearing loss	Speech and language delay, learning disability—32/34 had speech delay	n/a
Annunziata et al. [81], 2023	n/a	Maternal inheritance in ⅘ subjects; incomplete penetrance	n/a	n/a	Developmental delay	Intellectual disability	n/a	Epileptiform discharge while sleeping or falling asleep	n/a	n/a	n/a	n/a
Williams et al. [83], 2019	Alpha	Paternal inheritance of miR-873-5p variant; maternal inheritance of NRXN1 loss of function	n/a	n/a	n/a	n/a	n/a	n/a	n/a	n/a	n/a	n/a
Leblond et al. [36], 2019	alpha	none	yes	NR	NR	Intellectual disability	no	NR	NR	NR	NR	Congenital torticollis and dental carries
Uzunhan et al. [80], 2022	NR	alpha	yes	no	NR	NR	No	NR	Yes	NR	Yes	Macrocephaly, frontal bossing, bitemporal narrowing, wide forehead, long face, thin upper lip
Cameli et al. [82], 2021	NR	Other rare variants found unspecified	NR	Maternal history of mutation—no family history of ASD	Yes	NR	NR	Predominance of a slow background activity in the R temporal region	Yes, delayed with motor stereotypies (hand flapping); limited speech (four words)	Yes, manipulating materials for visual, acoustic, tactile stimulation	Yes—hyperactivity, short attention span	
Ishizuka et al. [77], 2020	Alpha	NR	NR	Maternally inherited	NR	Yes, No	NR	NR	NR	NR	NR	[ODD (oppositional defiant disorder)], [Depression, ADHD]

NR: not recorded.

## Data Availability

Not applicable.

## Supplementary Materials

The following supporting information can be downloaded at: https://www.mdpi.com/article/10.3390/jcm13072067/s1, Figure S1: Location of neurexins and their binding partners, neuroligins, in the synapse. Several neurexin–neuroligin pathway proteins are shown as well as synaptic vesicle-binding proteins. NMDAR, N-methyl-Daspartate receptor; mGluR5, metabolic glutamate receptor 5; PSD-95, post-synaptic density protein 95; Shank, SH3 and multiple ankyrin repeat domains protein. Taken from [29] under the terms of the Creative Com-mons Attribution License, which permits unrestricted use, provided the original author and source are credited.
