# Supplementary material for: Landscape of NRXN1 Gene Variants in Phenotypic Manifestations of Autism Spectrum Disorder: A Systematic Review
Source: J Clin Med. 2024 Apr 2;13(7):2067. doi: 10.3390/jcm13072067 (PMC11012327; doi:10.3390/jcm13072067)
Supplement: Supplementary file 1 [file jcm-13-02067-s001.zip › jcm-2907357-supplementary.pdf]

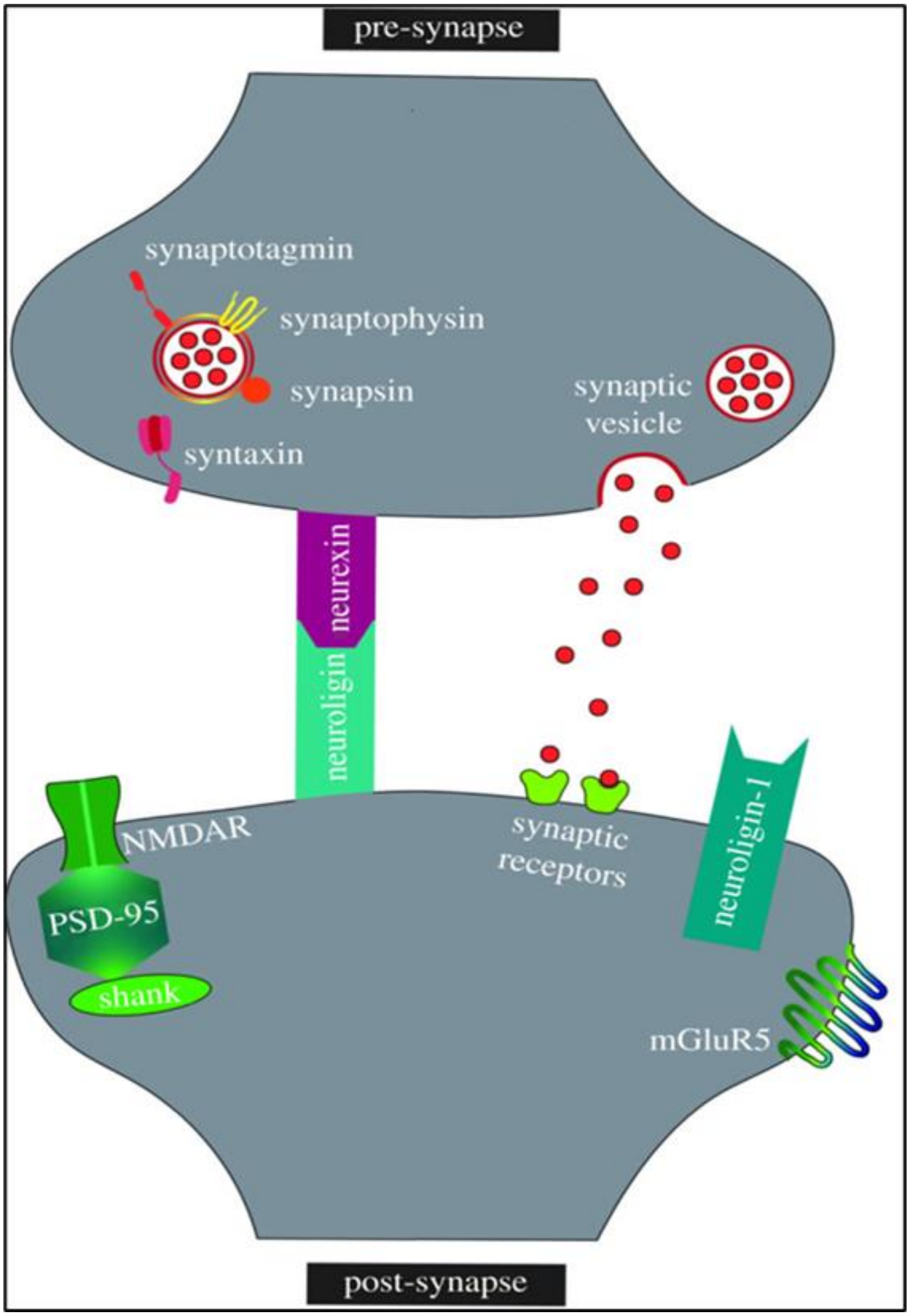

Supplementary Figure 1. Location of neurexins and their binding partners, neuroligins, in the synapse. Several neurexin–neuroligin pathway proteins are shown as well as synaptic vesicle-binding proteins. NMDAR, N-methyl-D-aspartate receptor; mGluR5, metabolic glutamate receptor 5; PSD-95, post-synaptic density protein 95; Shank, SH3 and multiple ankyrin repeat domains protein. Taken from [29] under the terms of the Creative Commons Attribution License, which permits unrestricted use, provided the original author and source are credited.
